# Radiosensitizing effect of curcumin-loaded lipid nanoparticles in breast cancer cells

**DOI:** 10.1038/s41598-019-47553-2

**Published:** 2019-07-31

**Authors:** Luigi Minafra, Nunziatina Porcino, Valentina Bravatà, Daniela Gaglio, Marcella Bonanomi, Erika Amore, Francesco Paolo Cammarata, Giorgio Russo, Carmelo Militello, Gaetano Savoca, Margherita Baglio, Boris Abbate, Giuseppina Iacoviello, Giovanna Evangelista, Maria Carla Gilardi, Maria Luisa Bondì, Giusi Irma Forte

**Affiliations:** 1 Istituto di Bioimmagini e Fisiologia Molecolare-Consiglio Nazionale delle Ricerche (IBFM-CNR), Cefalù, (PA) Italy; 2 SYSBIO Centre of Systems Biology, University of Milano-Bicocca, Milano, Italy; 3 Istituto per lo Studio dei Materiali Nanostrutturati-Consiglio Nazionale delle Ricerche (ISMN-CNR), Palermo, Italy; 4 Medical Physics Department, ARNAS-Civico Hospital, Palermo, Italy; 5 Radiation Oncology, ARNAS-Civico Hospital, Palermo, Italy; 6 Department of Medicine and Surgery, University of Milano-Bicocca, Monza, Italy

**Keywords:** Radiotherapy, Systems biology

## Abstract

In breast cancer (BC) care, radiotherapy is considered an efficient treatment, prescribed both for controlling localized tumors or as a therapeutic option in case of inoperable, incompletely resected or recurrent tumors. However, approximately 90% of BC-related deaths are due to the metastatic tumor progression. Then, it is strongly desirable to improve tumor radiosensitivity using molecules with synergistic action. The main aim of this study is to develop curcumin-loaded solid nanoparticles (Cur-SLN) in order to increase curcumin bioavailability and to evaluate their radiosensitizing ability in comparison to free curcumin (free-Cur), by using an *in vitro* approach on BC cell lines. In addition, transcriptomic and metabolomic profiles, induced by Cur-SLN treatments, highlighted networks involved in this radiosensitization ability. The non tumorigenic MCF10A and the tumorigenic MCF7 and MDA-MB-231 BC cell lines were used. Curcumin-loaded solid nanoparticles were prepared using ethanolic precipitation and the loading capacity was evaluated by UV spectrophotometer analysis. Cell survival after treatments was evaluated by clonogenic assay. Dose–response curves were generated testing three concentrations of free-Cur and Cur-SLN in combination with increasing doses of IR (2–9 Gy). IC_50_ value and Dose Modifying Factor (DMF) was measured to quantify the sensitivity to curcumin and to combined treatments. A multi-“omic” approach was used to explain the Cur-SLN radiosensitizer effect by microarray and metobolomic analysis. We have shown the efficacy of the Cur-SLN formulation as radiosensitizer on three BC cell lines. The DMFs values, calculated at the isoeffect of SF = 50%, showed that the Luminal A MCF7 resulted sensitive to the combined treatments using increasing concentration of vehicled curcumin Cur-SLN (DMF: 1,78 with 10 µM Cur-SLN.) Instead, triple negative MDA-MB-231 cells were more sensitive to free-Cur, although these cells also receive a radiosensitization effect by combination with Cur-SLN (DMF: 1.38 with 10 µM Cur-SLN). The Cur-SLN radiosensitizing function, evaluated by transcriptomic and metabolomic approach, revealed anti-oxidant and anti-tumor effects. Curcumin loaded- SLN can be suggested in future preclinical and clinical studies to test its concomitant use during radiotherapy treatments with the double implications of being a radiosensitizing molecule against cancer cells, with a protective role against IR side effects.

## Introduction

Radiotherapy (RT) is a vital component of multimodal therapy for many types of cancer. For example, in Breast Cancer (BC) care, RT is considered an efficient treatment, prescribed as adjuvant therapy strategy combined with surgery for controlling localized tumors, as the last option for patients with inoperable cancers and also as the first choice in cases of incompletely resected or recurrent tumors after surgery^1^.

Overall BC women candidates for conservative surgery were also treated with RT to consolidate surgical treatment and success rate. According to the international BC treatment guidelines, the recommended total dose to be administered to BC patients is 50–60, with a fractionation of 2 Gy/die for 5 days/week^2^. Moreover, in some cases of high recurrence risk (i.e. ductal invasive and microinvasive carcinoma), a radiation *boost* of 10–20 Gy could be administered in order to improve local control. Nonetheless in a phase III study involving 2657 BC patients, the radiation *boost* after whole-breast irradiation had no effect on long-term overall survival, with the largest absolute benefit in young patients^3–5^. In addition, approximately 90% of BC-related deaths are due to the metastatic progression of the tumor^6^. Then, it is strongly desirable to improve the tumor radiosensitivity in order to further reduce cancer recurrence. In this sense, one successful strategy could be to combine RT schedules with targeted therapies by using molecules with synergistic and radiosensitizing action.

Among these molecules, characterized by specific anti-cancer actions, the nutraceutical compounds often available in medicinal plants, have recieved a recent strong interest by several authors. Among these, a prominent role is reserved to the *Curcuma Longa* plant for its potentially anti-cancer multitarget effect and its active compounds, which are represented by curcuminoids. The curcuminoids belong to the polyphenol family, and are known to play key roles in the control of inflammation and oxidative stress in many conditions^7^. In particular, among these compounds, curcumin is known to play multiple pleiotropic effects (i.e. anti-bacterial, anti-fungal, antiviral, anti-oxidant, anti-inflammatory), thanks to its ability to interact and regulate multiple molecular targets such as transcription factors (TFs), growth factors, kinases, pro-inflammatory cytokines, adhesion molecules, etc. In turn, literature data highlight its potential therapeutic effects useful for the prevention and treatment of various diseases, including BC^8^.

On the other hand, thanks to its low toxicity and great anti-inflammatory effects, curcumin has recently been studied as a radiosensitizing molecule against tumor cells and radioprotective agent for healthy tissues^9^. In particular, the radiosensitizing role is supposed to be related to curcumin-induced inhibition of NF-KB, AP-1 and STAT3 transcription factors (TFs), highly constitutively expressed in numerous cancers and also transiently induced after ionizing radiation (IR) exposure. Moreover, curcumin is described to induce radiosensitization through the inhibition of such genes involved in several processes such as survival (Bcl-2, Bcl-XL), proliferation (COX-2, cyclin D1, and c-Myc), angiogenesis (VEGF and IL-8), invasion (MM9) and metastasis (ICAM-1, VCAM-1, ELAM-1). Overall, all these factors could contribute to the development of radioresistance in neoplastic cells^10,11^.

On the other hand, literature data report that RT treatments combined with curcumin inhibit proliferation and induce apoptosis of neoplastic cells, significantly improving the effects of RT, thanks to the suppression of cyclin D1, implicated in the G1/S cell cycle transition^12^ or by enhancing the effect of RT to arrest cell cycle in G2/M^13^. In this phase, cells are about three times more radiosensitive because, once they pass the G2 checkpoint, they are unable to repair DNA damage. Interestingly, the effect of radiosensitization is also mediated by increasing the intrinsic and extrinsic apoptosis induced by ionizing radiation (IR), through an increase of p53 expression by the MDM2 down-regulation mediated by Akt.^14^. Regarding the intrinsic apoptosis process, curcumin has been found to increase the mitochondrial Ca^2+^ level and Reactive oxygen species (ROS) production, resulting in an increased permeability of the mitochondrial membrane, allowing cytochrome C to translocate into the cytosol and bind Apaf-1 to form the apoptosome complex which triggers the caspase cascade (by activation of caspase-9) and subsequent cell death^15^. On the other hand, curcumin induces extrinsic apoptosis of neoplastic cells by increasing the expression levels of the “death activators” Fas ligand, TRAIL and TNF-α and, consequently, causing the activation of caspases 8 and 3^16^.

Although tumor cell radiosensitization is considered a promising idea to fight cancer diseases, it is equally important to reduce IR toxic effects in healthy tissues surrounding tumors. So, since one of the most prominent cancer pathogenic factors are of an inflammatory nature, it follows that the curcumin anti-inflammatory powerful effects could down regulate inflammation and ROS production, through to the inhibition of NF-kB. This mechanism is also involved in the reduction of the RT induced fibrosis, driven by TGFβ which is also under the NF-kB transcription control, so that curcumin could even contribute in the reduction of normal tissue toxicities following RT^17^. Then, curcumin could play a key interesting role in radiosensitivity/radioresistance cell balance. However, considering its chemical characteristics, it is necessary to study new strategies to increase its absorption in cells. In particular, curcumin is a lipophilic and hydrophobic compound, and therefore insoluble in water and soluble in ethanol, dimethyl sulfoxide (DMSO) and acetone. It is poorly absorbed by the body, so much so that its serum concentrations after oral administration are particularly low. Curcumin stability is pH-dependent and in a basic environment it is degraded very quickly^8^. After oral administration, curcumin is metabolized by reduction and conjugation, producing metabolites vehicled by glucuronic acid and sulfate in plasma, whose biological activities are strongly reduced compared to that of curcumin. In this study, to facilitate its absorption, we have trapped curcumin into a Solid Lipid Nanoparticle carrier (SLN), in order to realize a molecule having a better *in vivo* biodistribution and an improved bioavailability and stability.

Summarizing, the principal aim of this study was to evaluate the radiosensitizing ability of curcumin loaded SLN (Cur-SLN), in comparison to free curcumin (free-Cur) by using an *in vitro* approach on BC models. Here we used the MCF10A immortalized non tumorigenic mammary epithelial cell line and MCF7 (ER+/PR+/HER2−), MDA-MB-231 (ER−/PR−/HER2−) tumorigenic immortalized cell lines, characterized by different tumorigenic aggressive phenotypes^18^. In addition, we also reported in a descriptive way, transcriptomic and metabolomic profiles and a quantification of oxidative stress induced by Cur-SLN treatments, highlighting intracellular networks modulated sustaining the radiosensitizing Cur-SLN role in irradiated breast cell lines.

We trust that all the data here reported may encourage the use of curcumin-loaded SLN in preclinical studies to test the combination with the radiotherapy treatment plans and its application in clinical trials.

## Methods

### Curcumin nanoparticles preparation

Empty and curcumin-loaded nanoparticles were prepared using an ethanolic precipitation technique followed by ultraturrax homogenization. Briefly, 118 mg of Precirol ATO was heated at 5–10 °C above its melting point (56 °C). In order to obtain drug-loaded nanoparticles, curcumin (20 mg) was added, under mechanical stirring, to the melted lipid phase. An ethanolic solution (2 ml) containing Pluronic F68 (200 mg) and then the cationic surfactant dimethyldioctadecyl-ammonium bromide (30 mg) was added to the melted lipid phase. Finally, the resulting warm organic solution was dispersed into the bidistilled water (100 ml) at 80 ± 1 °C and homogenized by Ultraturrax T25 (IKA Labortechnik, Germany) at 17,500 rpm for 10 min. The obtained emulsion was quickly cooled using an ice bath for 30 min and then purified by exhaustive dialysis (12,000–14,000 Dalton cutoff membranes, Spectra/Por®, USA). After purification, to prevent any nanoparticle aggregation, a cryoprotector (trehalose) to nanoparticle suspensions was added, using nanoparticles:cryoprotector ratio of 1:2 (w/w). Finally, curcumin nanoparticles were freeze-dried by using a lyophilizer (FreeZone® 4.5 Freeze Dry System, Labconco Corporation, MO, USA) and stored at room temperature for successive characterization.

### Particle size analysis and Zeta-potential measurements

The hydrodynamic diameter (z-average) of empty and curcumin loaded nanoparticles was determined by Photon Correlation Spectroscopy (PCS) by using a Zetasizer Nano ZS (Malvern Instrument Ltd, UK), as reported in literature^19^. The samples were diluted in bidistilled water until the appropriate concentration and then the measurements were carried out at 25 ± 1 °C, at a fixed angle of 173° (NIBS = non-invasive backscattering detection) in respect to the incident beam. Each sample was kept in a cuvette and analyzed in triplicate. Zeta potential (ζ-potential) values were measured at 25 ± 1 °C using samples appropriately diluted in bidistilled water. The instrument setting conditions were equal to those described above for size measurements. Each sample was analyzed in triplicate.

### Drug loading (DL%) and Entrapment Efficiency (EE%) determination

Curcumin loading capacity was evaluated by UV spectrophotometer analysis. The nanoparticles were solubilized using organic mixtures until the appropriate concentrations [dichloromethane/acetonitrile/ethanol 4/4/2 (v/v/v)], filtered through 0.45 μm cellulose acetate membrane filters (VWR, Milan, Italy) and analyzed by UV spectrophotometer (UV-1800 Shimadzu, Kyoto, Japan), at 423 nm. The peak areas were compared with the one obtained by analyzing standard solutions of curcumin at known concentrations. The straight-line equation was: y 132.8x + 0.0084 and the linear regression value was: r2 = 0.9992. The linearity of the method was studied for curcumin concentrations in the range of 1–20 μg/ml. DL% results were expressed as the weight percent ratio between the curcumin loaded and the total dried sample weight. Entrapment efficiency (EE%) was expressed as the weight percent ratio between the amount of curcumin entrapped into nanoparticles and the total amount of curcumin used to prepare the particles. Below, the equations used for DL% and EE% determination are reported.

$${\rm{DL}}( \% )={\rm{Wdrug}}/{\rm{WNp}}\times 100$$

Wdrug represents the amount of curcumin entrapped into the nanoparticles and WNp represents the weight of the drug-loaded nanoparticles.

$${\rm{EE}}( \% )={\rm{Wf}}/{\rm{Wi}}\times 100$$

Wf represents the amount of curcumin entrapped and Wi represents the amount of curcumin used to prepare the curcumin-loaded nanoparticles.

### Cell cultures and intracellular and extracellular concentrations of free-Cur and Cur-SLN

The human non-tumorigenic breast epithelial MCF10A cell line and the human breast adenocarcinoma MCF7 and MDA-MB-231 cell lines, were purchased from the American Type Culture Collection (ATCC, Manassas, VA) and cultured according to the ATCC’s specifications. Cells were maintained in an exponentially growing culture condition in an incubator at 37 °C in a humidified atmosphere (95% air and 5% CO_2_) and were routinely subcultured in 25-cm^2^ (T25) and 75-cm^2^ T75) standard tissue culture flasks. Cell lines were cultured in the presence and in the absence of 2.5 µM of free- Cur or Cur-SNL for 24 hrs. At the end of the stimulation, cells and supernatants were collected to assess the intracellular and extracellular content of curcumin by UV analysis. In particular, the supernatants were sucked from the wells and then collected by centrifugation at 1,300 rpm for 10 min. Cells were detached from the wells by tripsin, washed with PBS and stored as dry pellets at −80 °C.

### Clonogenic survival assay and IC50 determination

Cell survival after treatments was evaluated by clonogenic assay performed as previously described^20,21^. Briefly, 48 hours before treatments cells were detached, counted by haemocytometer and re-plated in triplicate at opportune densities in 6-well plates to assay the surviving fraction (SF). As control (basal), untreated cells were seeded in the same conditions in order to evaluate the plating efficiency (PE). All cell survival data post-treatment were normalized to the untreated sample. Colonies were allowed to grow under normal cell culture conditions for 10–15 days after treatments and then were fixed and stained with 50% methanol and 0.5% crystal violet (both from Sigma-Aldrich, St. Louis, MO, USA). Colonies with more than 50 cells were counted manually under a Zeiss Axiovert phase-contrast microscope (Carl Zeiss, Göttingen, Germany) and also automatically with a computer-assisted methodology^22^.

Empty SLN has been tested for cellular toxicity on the three cell lines under study using the concentrations of 2.5, 5, 10, 25, 50 and 100 μM and showing not to be toxic, as no significant reduction of cell survival has been observed even at the higher concentration of 100 μM (data not shown). In order to obtain the IC_50_ value of free-Cur and Cur-SLN for each breast cell line, which represents the concentration required to obtain the 50% of *in vitro* inhibition, cells were treated in triplicates using a wide range of free-Cur and Cur-SLN concentrations (2.5, 5, 10, 25, 50 and 100 μM) in 6-well plates. 24 hrs after treatments, the medium was replaced and the cells were maintained in standard growth conditions until processing according to the clonogenic assay protocol. The IC_50_ was calculated from the two drug dose–response curves obtained by the increase of concentrations of free-Cur and Cur-SLN, using a 2nd order polynomial fitting analysis. Moreover, dose-response curves were generated by means of the 2nd order polynomial fitting of FS% *vs* increasing IR dose for each concentration of free-Cur and Cur-SLN used.These curves were used to obtain the Dose Modifying Factor (DMF), arbitrarily calculated at the SF of 50% (i.e. the relative dose of irradiation required to obtain the isoeffect of SF = 50% with radiation treatment alone respect to combined treatments with a defined concentration of free-Cur or Cur-SLN^23^.

### Radiation treatments

A medical linear accelerator, Siemens Primus (Siemens Medical Systems, Concord, CA, USA) emitting photon beams (X-rays) of 6 MV nominal energy, was used to perform cell irradiations. The Linac was calibrated under reference conditions defined by the International Atomic Energy Agency Technical Reports Series No. 398 “Adsorbed Dose Determination in External Beam Radiotherapy”^24^. The irradiation setup and the dose distribution were studied using the Pinnacle Treatment Planning System (Philips Medical Systems). Cell irradiations were carried out using doses of 2, 4, 6 and 9 Gy to evaluate the radiation efficacy through a dose-response curve.

### Oxidative stress quantification

A quantification of cellular oxidative stress has been performed measuring the intracellular reactive oxygen species (ROS) in the control (untreated), 2 Gy, 2 Gy + Cur-SLN, Cur-SLN samples of the three cell lines studied. The production of ROS was evaluated using 2′,7′-dichlorodiidrofluorescineacetate (DCFH-DA) (Molecular Probes, Eugene, OR). The DCFH-DA is de-esterified intracellularly and turns highly fluorescent 2′,7′-dichlorofluorescein upon oxidation, allowing a sensitive and rapid quantitation of oxygen reactive species in response to oxidative metabolism. 24 hours after treatments, cells were trypsinized, washed with PBS and incubated with 5 μM DCFH-DA reagent working solution according to manufacturing protocol. For consistency, 10^5^ cells were analyzed by fluorimeter for each determination performed in triplicate. The oxidation reaction was evaluated using the GloMax® Discover System (Promega) at the excitation wavelength of 475 nm and emission wavelength of 555 nm. The data shown are obtained from three independent experiments and are expressed as the mean ± standard error of the mean (SEM). The significance level respect to the control sample was set to *P < 0.05.

### Whole genome cDNA microarray expression analysis

We decided to evaluate only gene expression profiles (GEP) generated by the administration of Cur-SLN, as this formulation could have a clinic interest. Furthermore, we decided to evaluate the lowest dose/concentration combination (2 Gy/2.5 µM), a synergistic radiosensitizing treatment aims to obtain the maximum efficacy by administering the minimum doses of a drug and RI. Furthermore, it should be remembered that 2 Gy is the daily dose delivered in fractionated radiotherapy treatments.

To analyze GEP induced by 2.5 µM Cur-SLN in MCF10A, MCF7 and MDA-MB-231 cell lines treated with 2 Gy of photon beams, we used whole human genome 4 × 44 K cDNA microarray expression analyses. In particular, we analyzed GEP of the following configurations: MCF10A Cur-SLN 2 Gy normalized with MCF10A 2 Gy; MCF7 Cur-SLN 2 Gy normalized with MCF7 2 Gy; MDA-MB-231 Cur-SLN 2 Gy normalized with MDA-MB-231 2 Gy.

Total RNA extraction and its qualitative (expressed in term of RNA integrity number evaluation, RIN) and quantitative analyses were conducted as previously described^20,21^. Microarray experiments were performed according to the Agilent Two-Color Microarray-Based Gene Expression Analysis protocol (Agilent Technologies, Santa Clara, CA, USA). In turn, 6 replicates for each configuration assayed were performed. Statistical data analysis, background correction, normalization and summary of expression measures were conducted with GeneSpring GX 10.0.2 software (Agilent Technologies). Finally, genes were identified as being differentially expressed if they showed a fold change (FC) of at least 1.5 with a p-value < 0.05 compared to irradiated cells of the same cell line analyzed, that were used as reference samples. The data discussed in this publication, in compliance with Minimum Information About a Microarray Experiment (MIAME) standards, have been deposited in the National Center for Biotechnology Information Gene Expression Omnibus (GEO)^25^ and are accessible through the following GEO Series accession number: GSE118061.

In addition, the GEPs obtained in this work were also analyzed by pathway analysis using the Database for Annotation, Visualization and Integrated Discovery (DAVID) network building tool that provides a comprehensive set of functional annotation tools for investigators to understand the biological meaning behind a long list of genes (https://david.ncifcrf.gov/tools.jsp). The most representative significantly changed networks and pathways were selected and analyzed.

### Metabolite profiling of cell culture samples

Metabolites from the same treatments described in the above transcriptomic section (2 Gy and 2.5 µM Cur-SLN) were extracted and analyzed by gas chromatography-mass spectrometry (GC-MS), as previously described^26^. Derivatization was performed using automated sample prep WorkBench instrument (Agilent Technologies). Dried polar metabolites were dissolved in 60 μl of 2% methoxyamine hydrochloride in pyridine (Pierce) and held at 40 °C for 6 hrs. After dissolution and reaction, 90 μl of MSTFA (N-Methyl-N-(trimethylsilyl) trifluoroacetamid) were added and samples were incubated at 60 °C for 1 h. GC/MS analysis was performed using 7200 accurate-mass Q-TOF GC/MS (Agilent Technologies) equipped with a 40-m DB-5MS capillary column operating under electron impact (EI) ionization at 70 eV. Samples (2 μl) were injected in a splitless mode at 250 °C, using helium as the carrier gas at a flow rate of 1 ml/min. The GC oven temperature was held at 100 °C for 2 min and increased to 325 °C at 10 °C/min.

GC/MS data processing was performed using Agilent Muss Hunter software and statistical analyses were performed using Mass Profile Professional (MPP) software (Musharraf *et al*., 2016). Relative metabolites abundance was carried out after normalization to internal standard d27-myristic acid and cell number.

### Ethics approval and consent to participate

This study does not require ethical approval and informed consent, as the research project uses commercial immortalized cell lines samples from the American Type Culture Collection (ATCC, Manassas, VA, USA).

## Results

### Curcumin loaded nanoparticles preparation and characterization

In literature is widely reported the potential application of curcumin but it was heavily limited in biomedicine because of its poor solubility in water^27–29^. The oral bioavailability of curcuminoids in healthy humans is markedly enhanced by micellar solubilisation but not further improved by simultaneous ingestion of sesamin, ferulic acid, naringenin and xanthohumol^30^. To date, to increase the bioavailability of curcumin several methods were developed. In the present work, empty and curcumin-loaded solid lipid nanoparticles (SLNs) were successfully prepared by using the ethanolic precipitation technique and then, stored at room temperature for successive characterization in terms of mean size, polydispersity index (PDI) and ζ-potential in bidistilled water (Table 1).

**Table 1:** Size, PDI, and ζ-potential of empty and curcumin loaded SLNs in bidistilled water.

Systems	Z-average (nm)	PDI	ζ-potential (mV) ± SD
empty SLNs	159.0	0.275	+44.9 ± 7.5
Curcumin SLNs	302.5	0.544	+41.4 ± 4.6

Table 1 shows that the curcumin presence inside the nanoparticle system caused an increase of the mean size (from 159 nm to 302 nm) and polidispersity index (PDI). The zeta potential values, instead, were similar in both the systems considered and they ensured an adequate repulsion of particles.

### Drug loading (DL%) and Entrapment Efficiency (EE%) determination

In order to investigate the amount of curcumin entrapped into the SLNs, UV spectrophotometrics analysis was performed, as reported in the Materials and Methods section. The drug loading value of SLNs prepared, expressed as weight percent ratio between entrapped curcumin and the total dried sample weight (DL% w/w) and the Entrapment Efficiency (EE%), expressed as the weight percent ratio between the amount of curcumin entrapped into SLNs and the total amount of curcumin used to prepare the particles, were 1.64% and 68.62% respectively.

### IC50 determination

In order to evaluate the cytotoxicity ability of free curcumin and curcumin-loaded SLN (Cur-SLN) in term of concentration that determined the 50% of growth inhibition (IC_50_), MCF10A, MCF7 and MDA-MB-213 cell lines were treated with increasing doses of free-Cur and Cur-SLN, ranging from 2.5 to 100 μM for 24 hours and subjected to long-term clonogenic assay.

Free-curcumin and Cur-SLN significantly inhibited the viability of MCF7 cells, which showed greater sensitivity to Cur-SLN than free-Cur, displaying IC_50_ values of 2.45 μM and 3.80 μM, respectively. The cytotoxic effect of Cur-SLN was comparable to that of free curcumin in MDA-MB-231 cells, where IC_50_ values were of 7.60 μM and 6.63 μM, respectively. Finally, MCF10A cells were more sensitive to Cur-SLN than to free curcumin, displaying IC_50_ values of 6.81 μM and 10.58 μM, respectively.

### Cell radiosensitization following combined treatments with free-Cur and Cur-SLN

To evaluate the radiosensitizing ability of Cur-SLN in comparison to free-Cur, we investigated the combined effects of each of these two molecules on BC cells exposed to different ionizing radiation (IR) doses.

MCF10A, MCF7 and MDA-MB-231 cell lines were treated with only RT with increasing photon doses of 2, 4, 6, 9 Gy or were irradiated after a pretreatment with 2.5, 5, 10 µM of free-Cur and Cur-SLN for 24 hrs. The surviving fraction (SF) values, obtained by clonogenic assay after treatments, were used in order to generate dose-response curves in absence or presence of a certain concentration of free-Cur or Cur-SLN for the three BC cell lines (See Additional file 1). This, in turn, led to define the dose modifying factor (DMF), arbitrarily calculated at the SF of 50% by means of a 2nd order polynomial fitting from the dose response curves (i.e. the relative dose of irradiation required to obtain the isoeffect of SF = 50% delivering the radiation treatment alone respect to combined treatments with different concentration of free-Cur or Cur-SLN) (Table 2)^23^. These values show an overall increasing radiosensitization effect, rising with the concentration of both the two drugs. However, the three cell lines show a different level of sensitivity to free-Cur and Cur-SLN. Indeed, the normal epithelial MCF10A showed to be the most sensitive to all the radiosensitizer combined treatments, in comparison with the other two tumorigenic cell lines, being more sensitive to the vehicle curcumin Cur-SLN for each concentrations tested. On the other hand, although a lesser radiosensitizer effect can be observed for the two tumorigenic cell lines, MCF7 resulted more sensitive to the treatment with increasing concentration of vehicled curcumin Cur-SLN, whereas MDA-MB-231 have proved to be more sensitive to free-Cur, reaching the maximum of radiosensitizer effect using 10 µM of free-Cur.

**Table 2:** Dose Modifying factors (DMF) for increasing concentrations of free-Cur and Cur-SLN.

		MCF10A	MCF7	MDA-MB-231
DMF_50__Photons/Photons + free-Cur	2,5 µM	1,29	0,99	0,93
	5 µM	1,44	0,92	1,08
	10 µM	2,28	1,68	1,72
DMF_50__Photons/Photons + Cur-SLN	2,5 µM	1,65	1,06	0,81
	5 µM	1,65	1,58	1,03
	10 µM	2,76	1,78	1,31

Table 2 displays the DMFs, arbitrarily calculated at the SF of 50%, by means of a polynomial fitting from the dose response curves.

**Figure 1:**
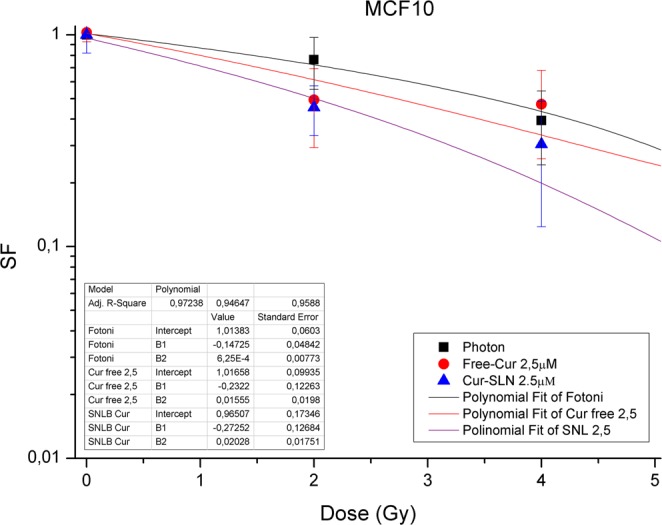
Dose-Response curves of MCF10A. Dose-Response curves of MCF10A cells subjected to increasing doses of IR with or without 2.5 µM of free-Cur and Cur-SLN.

**Figure 2:**
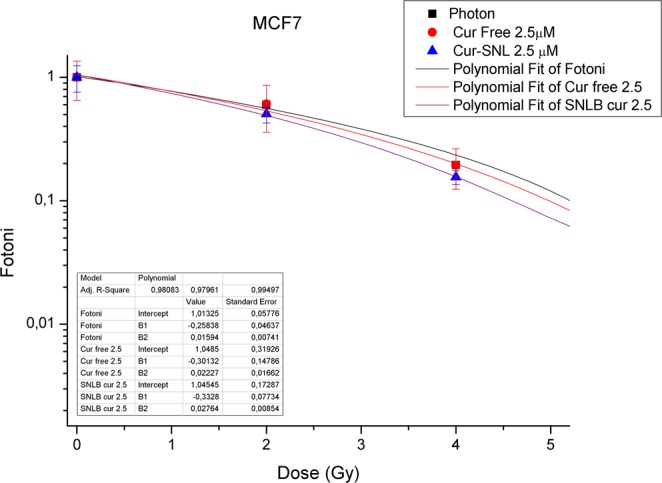
Dose-Response curves of MCF7. Dose-Response curves of MCF7 BC cells subjected to increasing doses of IR with or without 2.5 µM of free-Cur and Cur-SLN.

**Figure 3:**
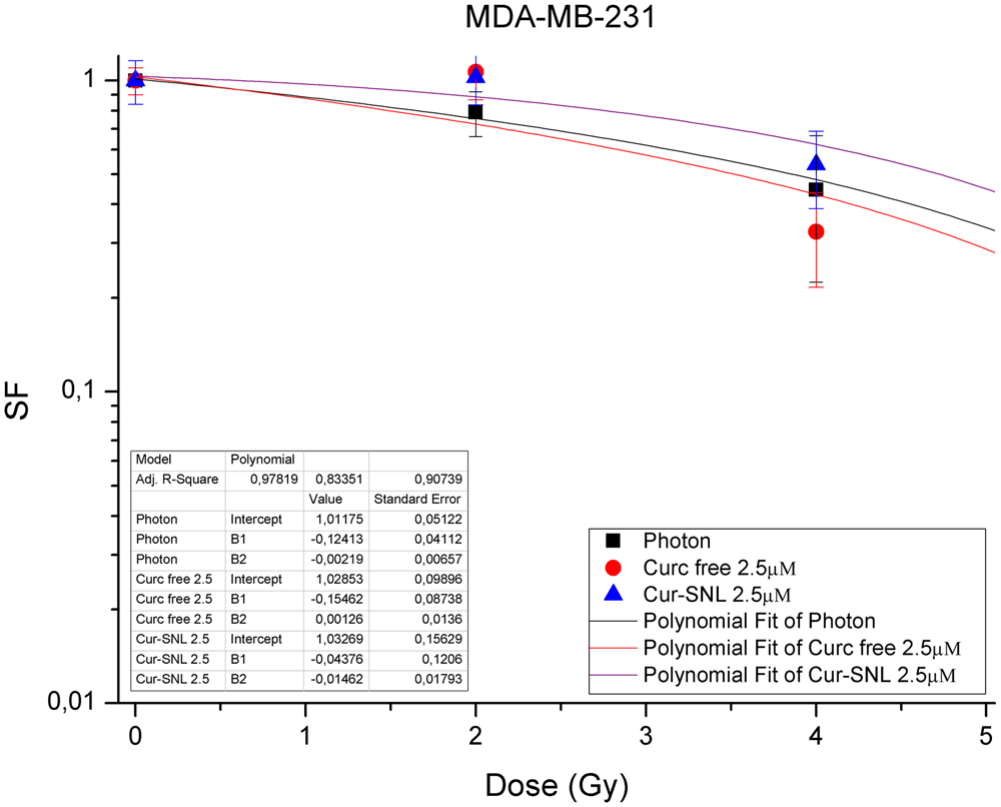
Dose-Response curves of MDA-MB-231. Dose-Response curves of MDA-MB-231 BC cells subjected to increasing doses of IR with or without 2.5 µM of free-Cur and Cur-SLN.

As we decided to study GEP and metabolomic changes caused by the administration of the minimum concentrations of Cur-SLN, Figs 1–3 display the relative effect of combined treatments with 2,5 µM of free-Cur and Cur -SLN respect to that deriving from the exclusive treatment with IR. A better response with the minimum dose of 2,5 µM of loaded Curcumin (Cur-SLN) is observable for MCF10A and MCF7, whereas MDA-MD-231 receives a protective effect by low concentrations of both free-Cur and Cur-SLN.

### Oxidative stress quantification

**Figure 4:**
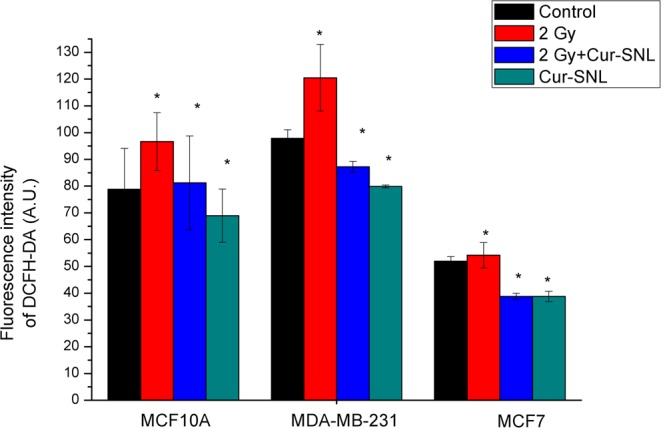
Determination of intracellular ROS production. Oxidative stress quantification performed measuring intracellular ROS levels with the DCFH-DA molecular probe in the MCF10A, MDA-MB-231 and MCF7 cell lines 24 hrs after treatments with 2 Gy, 2 Gy + Cur-SLN, Cur-SLN. The significance level respect to the control sample was set to *P < 0.05.

As shown in Fig. 4 the result of this assay shows and confirms the anti-oxidant properties of curcumin. Indeed, for all the three cell lines analyzed, a significant increase in the oxidative stress caused only by irradiation can be observed, whereas treatment with curcumin alone or in combination with IR is able to lower the level of cellular oxidative stress below the value of untreated cells (P < 0.05). Moreover, in the combined treatments, the level of oxidative stress is always significantly lower than that observed in samples subjected to irradiation only, demonstrating the curcumin efficacy in lowering the ROS levels produced following the administration of IR.

### Gene expression profiles and gene signatures after combined Cur-SLN and RI treatment

As above described we evaluated only GEP generated by Cur-SLN, as this formulation could have a clinic interest. Furthermore, we decided to evaluate the lowest dose/concentration combination (2 Gy/2.5 µM), a synergistic radiosensitizing treatment aims to obtain the maximum efficacy by administering the minimum doses of a drug and RI. Furthermore, it should be well-known that 2 Gy is the daily dose delivered in fractionated radiotherapy treatments.

Here, we reported GEP data obtained applying a Two-Color Microarray-Based Gene Expression Analysis (Agilent technologies) on MCF10A, MCF7 and MDA-MB-231 breast cell lines treated with 2.5 µM Cur-SLN and irradiated with photon beams using the IR dose of 2 Gy. In particular, comparative differential gene-expression analysis revealed that multiple deregulated genes (DEG) were significantly altered, by 1.5-fold or greater according to the specific experimental configuration. MCF10A cells treated with Cur-SLN changed the expression levels of 846 genes (189 down regulated and 657 up regulated). On the other hand, 1265 DEGs were selected in MCF7 BC cell line Cur-SLN treated and, among these, 752 were up regulated while 513 were down regulated. Finally, in MDA-MB-231 BC cell lines treated with Cur-SLN we selected 2301 DEGs (112 down regulated and 1179 up regulated).

Moreover, up and down regulated transcripts were selected and grouped according to their involvement in specific biological networks using Integrated pathway enrichment analysis with the DAVID tool^25^ and the top molecular pathways of DEG datasets were selected and reported in Tables 3–5.

**Table 3:** DAVID pathway analysis of the top pathways and related genes, deregulated after Cur-SLN treatment in MCF10 cell line.

Pathway analysis of GEP induced by SLNB-Cur in MCF10 non tumorigenic cell line					
Term		Genes count	%	P value	Genes
1	Lysine degradation	7	0.0071	0.0075125	KMT2C, WHSC1L1, ASH1L, WHSC1, SETD2, NSD1, SUV39H2
2	Transcriptional misregulation in cancer	12	0.0122	0.0255476	NFKBIZ, CCR7, RXRA, TP53, CDK9, FOXO1, MDM2, MLLT1, WHSC1, ZBTB16, JMJD1C, RUNX2

**Table 4:** DAVID pathway analysis of the top-5 pathways and related genes, deregulated after Cur-SLN treatment in MCF7 BC cell line.

Pathway analysis of GEP induced by SLNB-Cur in MCF7 BC cell line					
Term		Genes count	%	P value	Genes
1	Cytokine-cytokine receptor interaction	21	0.0145	0.0257	TNFRSF6B, TNF, OSMR, TNFRSF25, CSF1, LIFR, CD70, CX3CL1, CCL28, IL11RA, TGFB2, LIF, AMH, IL17A, IL20RB, CCR3, IL2RG, LTB, LTA, IL3RA, BMPR1A
2	Apoptosis	9	0.0062	0.0186	TNF, BAX, PIK3CD, CASP8, TP53, IL3RA, PIK3R1, AKT2, PIK3R2
3	Platelet activation	14	0.0097	0.0246	F2RL3, ADCY2, PIK3CD, ITGA2, APBB1IP, COL5A2, JMJD7-PLA2G4B, P2RX1, PRKACA, COL1A1, COL11A2, PIK3R1, PIK3R2, AKT2
4	Tyrosine metabolism	6	0.0041	0.0397	TYR, PNMT, MAOA, AOX1, HPD, AOC3
5	Glucagon signaling pathway	11	0.0076	0.0428	LDHB, CPT1B, ADCY2, PYGM, GCK, PPP3R1, PRKACA, ACACB, PPARGC1A, GCGR, AKT2

**Table 5:** DAVID pathway analysis of the top-5 pathways and related genes, deregulated after Cur-SLN treatment in MDA-MB-231 BC cell line.

Pathway analysis of GEP induced by SLNB-Cur in MDA-MB-231 BC cell line					
Term		Genes count	%	P value	Genes
1	FoxO signaling pathway	27	0.0094	0.0006	ATG12, FOXO1, TGFB2, IGF1R, PRMT1, FBXO25, CAT, EGF, AGAP2, PIK3R1, EGFR, GABARAPL2, IL6, PRKAB2, TGFBR2, SKP2, HOMER2, ATM, CDK2, BCL2L11, TNFSF10, PLK4, MAPK13, MAPK14, FBXO32, GADD45B, GADD45A
2	Cell cycle	24	0.0084	0.0021	ANAPC1, E2F2, E2F5, RBL1, SKP2, TTK, PRKDC, CDC20, WEE1, CDK2, ATM, TGFB2, MAD2L1, BUB1, TFDP2, YWHAQ, BUB1B, ANAPC7, ABL1, GADD45B, GADD45A, CCNA2, BUB3, TFDP1
3	TGF-beta signaling pathway	18	0.0063	0.0030	SMAD9, LTBP1, ROCK1, E2F5, FST, TGFBR2, RBL1, RPS6KB2, TGFB2, ID2, ID1, ZFYVE16, SMURF2, SMURF1, BAMBI, BMPR1A, BMP6, TFDP1
4	TNF signaling pathway	19	0.0066	0.0152	TRAF1, ICAM1, IL6, CSF1, CREB1, CXCL2, EDN1, NFKBIA, CREB5, CX3CL1, MMP14, CXCL10, RPS6KA5, BAG4, ATF4, MAPK13, MAPK14, MAP2K6, PIK3R1
5	Phosphatidylinositol signaling system	17	0.0059	0.0299	PRKCA, INPP1, PIK3C2A, PIK3C2B, SYNJ1, ITPKB, PI4K2B, PIP5K1A, DGKA, DGKE, PLCD4, INPP5E, MTMR8, INPP5D, PLCB1, INPP5B, PIK3R1

The result of this mapping revealed the involvement of a set of factors controlling cellular processes described as follows.

Summarizing, as displayed in Table 3, only 2 statistically relevant pathways were selected in MCF10A cell line treated with Cur-SLN. In particular, lysine degradation pathway is known to be mainly involved in the Acetyl-CoA production, modulated by curcumin through mitochondrial fatty acid oxidation^31^, whereas transcriptional misregulation in cancer signaling contains several factors known to be deregulated in cancer cells because of their key roles in controlling cell survival/death balance.

On the other hand, in MCF7 BC cell line Cur-SLN causes the deregulation of molecules belonging to apoptosis induction, inflammation signaling (sustained by cytokine and platelet activation pathways), tyrosine metabolism known to be involved in cellular stress response and in glucagon signaling pathway, recently described as modulated by curcumin to improve glucose tolerance (Table 4)^32–37^.

Finally, as displayed in Table 5, in MDA-MB-231 BC cell line the Cur-SLN treatment activate specific set of genes controlling cell cycle process, TGF-beta pathway, TNF signaling, phosphatidylinositol signaling and FoxO signaling pathway.

### Analysis of selected gene signatures Cur-SLN treatment induced

**Figure 5:**
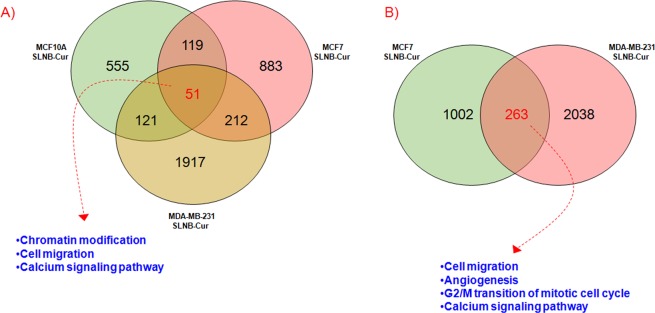
Venn diagrams of unique and shared differentially expressed genes by breast cell lines after combined treatments with IR/2,5 µM Cur-SLN. (**A**) A 51-gene signature of shared deregulate genes after Cur-SLN treatment was selected among MCF10A, MCF7 and MDA-MB-231 breast cell lines. The top-GO Biological processes were also displayed. (**B**) A 263-gene signature of common deregulated genes was selected for MCF7 and MDA-MB-231 BC cell lines and the top-GO biological processes regulated by these genes, were also reported in the figure.

Finally, in order to evaluate the unique and common DEG lists and cellular process modulated by these factors after Cur-SLN treatments in MCF10A, MCF7 and MDA-MB-231 cell lines, we performed Venn diagrams as shown in Fig. 5.

As displayed in Fig. 5A, common and unique DEGs were selected among all the configurations analyzed in this work. So, here we report an overall cell-dependent gene expression deregulation after Cur-SLN treatments. Moreover, as shown in Fig. 5A, 51 differentially expressed genes (51-gene signature) were shared among the three cell lines treated with Cur-SLN. Regarding these genes, GO Biological processes analysis conducted by the DAVID tool, revealed the activation of chromatin modification, cell migration and calcium signaling pathways. Finally, as displayed in Fig. 5B, we compared the list of DEGs of the two tumorigenic MCF7 and MDA-MB-231 BC cell lines treated with Cur-SLN, producing Venn diagrams (Fig. 5B). In particular, a 263-gene signature was selected and composed by DEGs involved in cell migration, angiogenesis, G2/M transition of mitotic cell cycle and calcium signaling pathways.

Moreover, in the Supplementary Files 1 and 2 we highlighted the gene expression value details of 51- and 263 gene signatures, respectively.

These data highlight the wide range of curcumin bioactivities, that could help to obtain successful treatment plans in combination with RT.

### Metabolomics change induced by Curcumin-Loaded SLN and irradiation treatments

To further understand better the effects induced by combined treatment of 2.5 µM Curc-SLN and irradiation with 2 Gy of photon beams in MCF10A, MCF7 and MDA-MB-231 human breast cell lines, we performed metabolomic analysis using gas chromatography/mass spectrometry (GC/MS) and liquid chromatography/mass spectrometry (LC/MS) systems.

**Figure 6:**
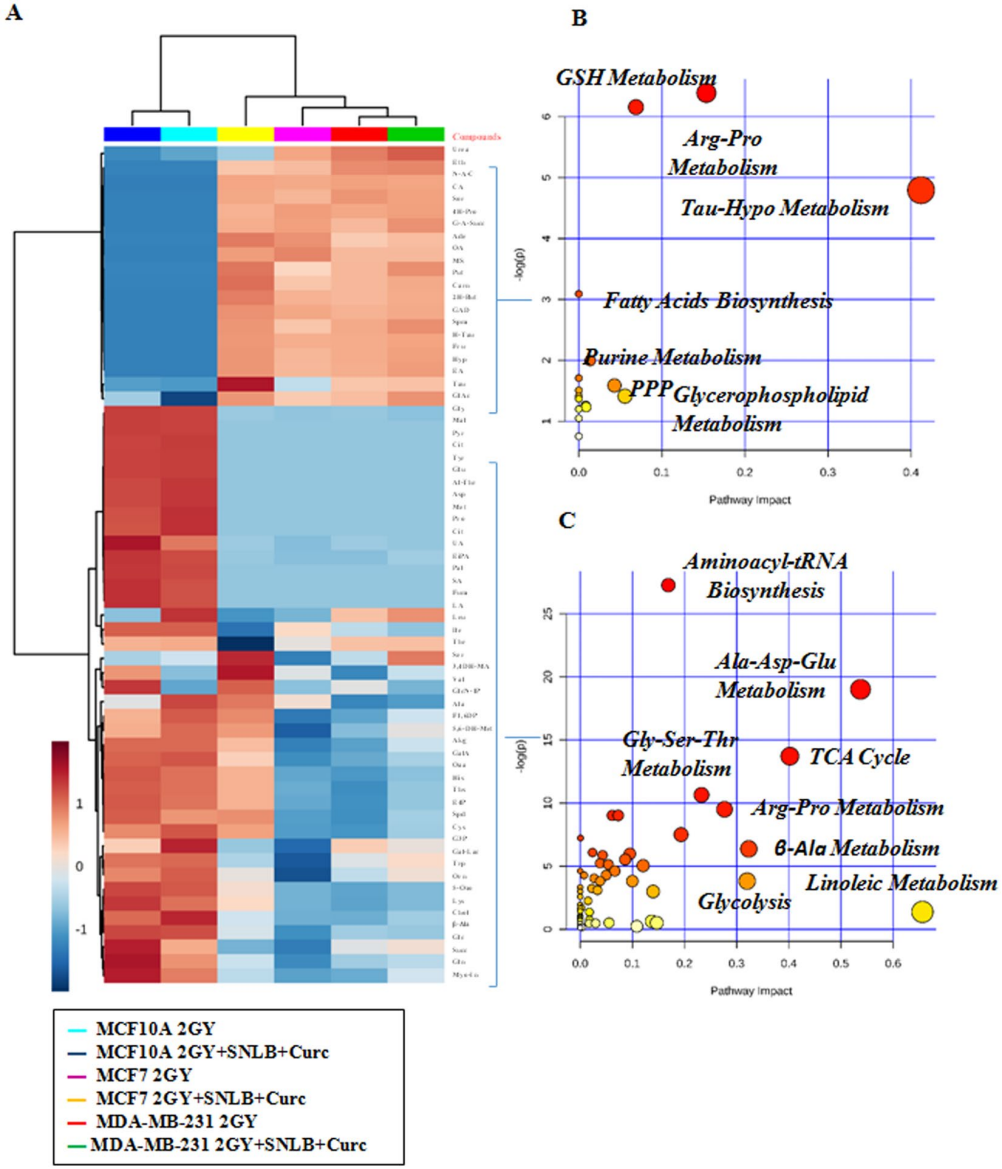
Comparative analysis of MCF10A, MCF7 and MDA-MB-231 by Hierarchical Clustering and untargeted enrichment plots. (**A**) Comparative analysis of MCF10A, MCF7 and MDA-MB-231 by Hierarchical Clustering. (**B**) Untargeted enrichment plot displaying increased metabolites levels in MCF7 and MDA-MB-231 under 2 Gy and 2 Gy/Cur-SLN treatments, respect to MCF10A under the same condition. (**C**) Untargeted enrichment plot displaying decreased metabolites levels in MCF7 and MDA-MB-231 under 2 Gy and 2 Gy/Cur-SLN treatments, respect to MCF10A under the same condition.

**Figure 7:**
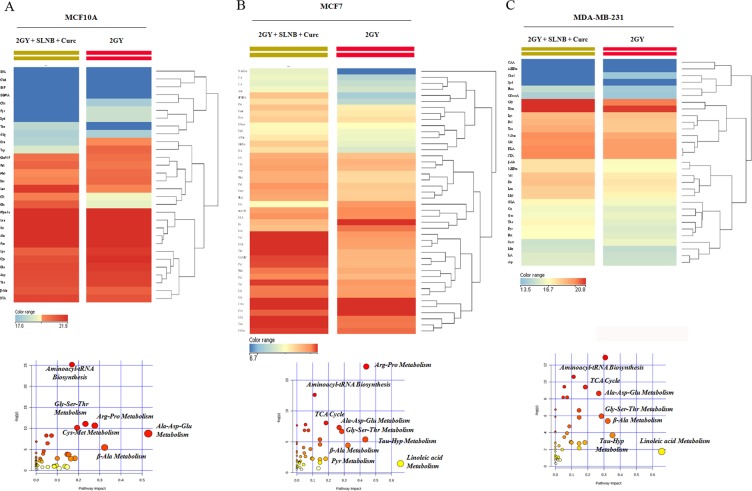
Hierarchical Clustering analysis and untargeted enrichment plots of MCF10A, MCF7 and MDA-MB-231 exposed to 2 Gy vs 2 Gy/Cur-SLN treatments. (**A**) Hierarchical Clustering analysis and untargeted enrichment plots of MCF10A, exposed to 2 Gy vs 2 Gy/Cur-SLN treatments. (**B**) Hierarchical Clustering analysis and untargeted enrichment plots of MCF7, exposed to 2 Gy vs 2 Gy/Cur-SLN treatments. (**C**) Hierarchical Clustering analysis and untargeted enrichment plots of MDA-MB-231, exposed to 2 Gy vs 2 Gy/Cur-SLN treatments.

The hierarchical clustering plot of metabolomic profiling revealed a similar metabolic phenotype between the two human breast cancer cell lines MCF7 and MDA-MB-231 under 2 Gy and 2 Gy/Cur-SLN treatments, as compared to MCF10A normal breast cell line (compare the right *vs* left side of the hierarchical clustering plot) (Fig. 6A). In particular, untargeted metabolic profiling, corresponding to the upper part of the hierarchical clustering plot, showed enrichment of increased of metabolites levels involved in anti-oxidant response such as: GSH metabolism, arginine and proline metabolism, pentose phosphate pathway (PPP) and taurine and hypotaurine metabolism, as well as in metabolites involved in fatty acids and nucleotide metabolism in MCF7 and MDA-MB-231 cell lines under 2 Gy and 2 Gy/Cur-SLN treatments, respect to MCF10A cells under the same conditions (Fig. 6B). On the contrary, the same cell lines (MCF7 and MDA-MB-231) under 2 Gy and 2 Gy/Cur-SLN treatments, showed significant decreased levels of metabolites involved in amino acids metabolism, TCA cycle and glycolysis as compared to MCF10A under the same conditions (see the lower part of the hierarchical clustering plot) (Fig. 6C). Moreover, the identification of the single branch clustering of MCF7 under 2 Gy/Cur-SLN treatments (yellow color) as compared to others (Fig. 6A) prompted us to perform a deeper statistical analysis in order to evaluate better the radiosensitising effect of loaded-curcumin in respect to irradiation only for each cell line studied. Statistical analysis of MCF10A exposed to 2 Gy *vs* 2 Gy/Cur-SLN treatments showed significant metabolites involved mostly in amino acids metabolism, highlighting increased significant levels of glucose and citrate in MCF10A exposed to the combined treatment of irradiation plus loaded curcumin (Fig. 7A). Similarly to MCF10A cells, comparison of MDA-MB-231 cancer cell line exposed to 2GY *vs* 2 Gy/Cur-SLN treatments showed significant metabolites involved both in amino acids metabolism and in anti-oxidant processes such as taurine and hypotaurine metabolism, fatty acids and TCA cycle (Fig. 7C). Finally, consistently with previous results observed in MDA-MB-231 cells, also MCF7 cells exposed to 2 Gy *vs* 2 Gy/Cur-SLN treatments showed significant increased metabolites involved in amino acids metabolism, in anti-oxidant action, fatty acids and TCA cycle probably also due to the activation of autophagy mechanism (Fig. 7B).

Therefore, taken together, these results showed that the effect of Cur-SNLB administration on breast cell lines exposed to irradiation is to exert a protective role against oxidative stress-induced by irradiation damage and to induce an antitumor effect through autophagy mechanism activation^38^.

## Discussion

The main aim of this research study was to evaluate the radiosensitizing effects mediated by Cur-SLN in comparison to that generated by free-Cur, using an *in vitro* approach on three immortalized breast cell lines: the epithelial non-tumorigenic MCF10A cell line and the two tumorigenic MCF7 (Luminal A: ER+/PR+/HER2−), and MDA-MB-231 (triple-negative: ER−/PR−/HER2−) cell lines. Specifically, these cell lines were subjected to single or combined treatment using four doses of IR (2, 4, 6 and 9 Gy) and 6 concentrations of free-Cur or Cur-SLN (2.5, 5, 10, 25, 50 and 100 μM). Empty SLN has been also tested for cellular toxicity on the three cell lines under study using the same above reported concentrations, showing it not to be toxic. The IC_50_ evaluation showed that all the cell lines tested are sensitive to treatment with free and curcumin loaded-SLN. However, the MCF7 tumorigenic cell line showed a strong sensitivity to treatment with Cur-SLN and free-Cur, compared to the other two cell lines analysed, with IC_50_ values of 2.47 μM and 3.81 μM, respectively. On the other hand, the two cell lines MCF10A and MDA-MB-231 displayed lower levels of sensitivity to both the molecules. In particular, while in MDA-MB-231 cells the cytotoxic effect of Cur-SLN was very similar to that of free curcumin, with IC_50_ values of 7.62 μM and 6.62 μM, respectively, the MCF10A were proved to be more sensitive to Cur-SLN than free curcumin by finding IC_50_ values of 6.74 and 10.56 μM respectively.

Based on the IC_50_ values, we tested the radiosensitizing effect of free-Cur and Cur-SLN, used at concentrations of 2.5, 5, 10 μM, in combination with conventional radiotherapy treatment with increasing doses of 2, 4, 6, 9 Gy, in order to generate dose/response curves for all the treatment configurations tested.

The radiosensitizing effect was determined by calculating the dose modifying factor (DMF), arbitrarily obtained at the SF of 50% (i.e. the relative dose of irradiation required to obtain the isoeffect of SF = 50% using the radiation treatment alone respect to combined treatments with different concentration of free-Cur or Cur-SLN), in order to highlight the capacity of enhancing tumor cells killing by the combined treatments in respect to irradiation only^23^.

In other terms, a DMF value of 2, means that the isoeffect could be obtained by the combined treatment with the half of IR dose, respect to the dose necessary using IR only.

Overall, the DMFs value reported in Table 2 highlight a general increasing radiosensitization effect, rising with the concentration of both the two drugs, nonetheless differences can be identified in the sensitivity of the three cell lines to free-Cur and Cur-SLN.

Indeed, the normal epithelial MCF10A received a strong radiosensitizer effect by the combined treatments, although they were more sensitive to the vehicle curcumin Cur-SLN for each concentrations tested. This means that further studies are needed to understand if the use of curcumin as radiosensitizer could increase normal tissues toxicity post-irradiation. However, it should be noted that normal tissue complications (NTCP) are of inflammatory nature and the powerful anti-inflammatory effects of curcumin could reduce these toxicities *in vivo*, reducing the inflammation and the reactive oxygen species production, thanks to the down-regulation of NF-kB. This mechanism is also involved in the reduction of fibrosis induced by radiotherapy, in which a key role is played by TGFβ^17^. On these aspects, a preclinical study evaluated the adverse effects of cutaneous toxicity that occur following exposure to IR^17^, evaluating a possible protective role exerted by curcumin. In this case, curcumin was administered intragastric and intraperitoneally in C3H/HeN mice, 5 days before RT or 5 days after RT. Skin damage was assessed at 15–21 days (acute cutaneous toxicity) and at 90 days (chronic cutaneous toxicity) following a single dose of 50 Gy of radiation in the posterior leg of each mouse. The results showed that curcumin, administered before or after radiotherapy, markedly reduces acute and chronic cutaneous toxicity, significantly decreasing the expression of inflammatory IL-1, IL-6, IL-18, IL-1Ra and fibrogenic (TGF-β) cytokines in irradiated skin and muscles.

On the other hand, although a lesser radiosensitizer effect can be observed for the two tumorigenic cell lines, MCF7 resulted more sensitive than MDA-MB-231 to the treatment with increasing concentration of vehicled curcumin Cur-SLN, reaching a DMF value of 1,78 using 10 µM of Cur-SLN. Instead, MDA-MB-231 were more sensitive to free-Cur, reaching the maximum of radiosensitizer effect with 10 µM of free-Cur (DMF = 1,72). However, a clinical administration of curcumin is likely in the vehicle form, then the aggressive triple negative MDA-MB-231 BC cell line could receive a radiosensitization effect by Cur-SLN, as the DMF value is 1.38 with 10 µM of Cur-SLN. Thus, the possibility to use drug/IR combined treatments permits to increase the tumor control probability (TCP) even for radioresistant tumors such as the triple negative BC.

Furthermore, in Figs 1–3 the efficacy of combined treatments is compared using the minimum concentration of 2,5 µM of free-Cur and Cur -SLN in respect to that deriving from the exclusive treatment with IR. A better response with the minimum dose of 2,5 µM of loaded Curcumin (Cur-SLN) is observable for MCF10A and MCF7 cells, whereas MDA-MB-231 receives a protective effect by low concentrations of both free-Cur and Cur-SLN.

Moreover, in order to highlight the action mechanism of Cur-SLN and to confirm the anti-oxidant properties of Cur-SLN, a quantification assay of oxidative stress induced by the treatments has been performed (Fig. 4). On the three cell lines analyzed, a significant increase in oxidative stress caused by only irradiation can be observed, whereas treatment with Cur-SLN alone or in combination with IR administration is always able to lower the level of cellular oxidative stress significantly below the values of untreated cells, demonstrating the curcumin efficacy in lowering the ROS levels produced following the administration of IR.

In addition, we carried out a “omic” study (transcriptomic and metabolomic), in order to explore better this new formulation (Cur-SLN) action mechanism in producing a radiosensitivity effect on the three cell lines analyzed. This is thanks to its formulation which facilitates its transport in an aqueous solution, it could be administered in preclinical or clinical application. Moreover, we only compared the effects of lower IR doses/drug concentrations, since the combination with a radiosensitizer should, hopefully, allow the use of lower IR doses maintaining the isoeffect of therapeutic efficacy and reducing the side effects to the minimum. In addition, 2 Gy is the daily dose administered in fractionated treatments with external beams. Therefore, for the “omic” study the comparisons considered were: 2 Gy *vs* 2 Gy + 2.5 μM Cur-SLN, for each cell line tested, analyzed with “whole genome microarray” approach and “two-color” staining method. The comparative gene expression analysis of MCF10A, MCF7 and MDA-MB-231 cells revealed that a large number of genes were deregulated (DEG) from treatment with Cur-SLN with a fold change (FC) ≥ 1.5. Below are the details of the genes deregulated in the three cell lines: 1) MCF10A: 846 DEG (189 down-regulated and 657 up-regulated); 2) MCF7: 1265 DEG (513 down-regulated and 752 up-regulated); 3) MDA-MB-231: 2301 DEG (112 down-regulated and 1179 up-regulated).

Figure 5 shows the detail of deregulated genes specific or common to the three cell lines analyzed, using a Venn diagram. As shown, the molecular response to Cur-SLN treatment is predominantly cell-line dependent, since most of the deregulated genes are specific of each cell line (MCF10A: 555 specific DEG, MCF7: 883 specific DEG, MDA-MB-231: 1917 specific DEG). On the other hand, the common response of the three cell lines to Cur-SLN treatment is determined by a signature of 51 genes, while the tumorigenic ones (MCF7 and MDA-MB-231) share a specific signature of 263 genes.

Thanks to the use of the DAVID tool, these deregulated genes have been grouped, based on their involvement in some biological networks and cellular processes. This approach has allowed to better characterize the unique and common molecular response among the analyzed cell lines. Specifically, in the non-tumorigenic MCF10A cell line, the specific molecular response to Cur-SLN treatment is characterized by the statistically significant deregulation of “Transcriptional misregulation in cancer”, which involves several key factors altered in neoplastic cells and “lysine degradation process” (Table 3), the latter involved in acetyl-CoA production, modulated by curcumin through mitochondrial oxidation of fatty acids^31^.

On the other hand, in MCF7 cell line the Cur-SLN treatment resulted in the deregulation of molecules involved in inflammatory processes, induction of apoptosis, tyrosine metabolism which is implicated in the response to cellular stress and glucagon signaling pathway, which is in turn regulated by curcumin in response to low glucose levels (Table 4)^31–37^. Finally, in MDA-MB-231 cells, deregulated genes involved in the cell cycle, in the TGF-β and in the TNF pathway, in the pathways of phosphatidylinositol and FoxO were identified (Table 5). Regarding the TGF- pathway, several authors reported that curcumin inhibits cell proliferation and invasion by preventing TGF-β1-induced phosphorylation of Smad2, ERK1/2, p38MAPK, resulting in down-regulation of MMP metalloproteinases-9, thus limiting the effect of cellular invasion^34,39^. Furthermore, it is also reported in the literature that curcumin is also able to trigger the extrinsic apoptotic pathway, allowing the binding of TNF-α and Fas Ligand “death activators” to their corresponding cell surface receptors that, by the activation of caspase-8, induce the caspase cascade^40^. In particular, these anti-tumor actions are largely modulated by curcumin by the negative regulation of various growth factors, inflammatory cytokines, transcription factors (TF), protein kinases and other oncogene molecules. In this context, the phosphatidylinositol-3-kinase (PI3K)/protein kinase B (AKT) pathway, usually activated in onset and neoplastic development, is inhibited by curcumin. This process could constitute a key molecular target for anti-cancer therapies as it represents a negative regulator of tumor progression processes and metastasis^32,41,42^.

Several studies have found that curcumin is also involved in the regulation of the FoxO pathway implicated in multiple cellular processes, such as cell cycle arrest, apoptosis, DNA repair, glucose metabolism and oxidative stress response. In particular, Fox TFs are negatively regulated by the AKT survival signal, as normally phospho-Akt prevents the nuclear localization of FOX and plays a key role in regulating its activity. Furthermore, tetrahydrocurcumin (THC), a metabolite of curcumin, regulates the oxidative stress response through FOXO, promoting its nuclear translocation and regulating, therefore, the expression of several genes involved in apoptosis, in cell cycle progression, in DNA repair, in oxidative stress, in the control of muscle growth, as well as in cell differentiation and glucose metabolism. Based on these findings, FOXO TF could be used as potential tumor suppressors^43–48^.

Similar molecular findings have been described in an *in vivo* study which evaluated the radiosensitizing effect of curcumin on nude mice inoculated with colon cancer HCT 116 cells, performed by the group of Kunnumakkara^49^. This study evaluated the effect of the combined administration of IR (4 Gy, twice a week) and curcumin administered orally 1 hour before IR exposure (1 g/kg). The effect was compared with a control group and two groups corresponding to the two treatments performed in single. The results showed that the tumor volume of mice that received combined treatment was significantly reduced compared to that of the control group of mice and those treated with curcumin alone or with radiation alone. Furthermore, it has been found that curcumin enhances the effect of RI, inhibiting the proliferation of neoplastic cells and generating a significant reduction of the Ki-67 cell proliferation marker and of CD31 (tumor density microvase marker) in tumor tissues, compared to the group that received only RIs. The same study showed a decrease in the expression levels of COX-2, VEGF, MMP-9, Cyclin D1, c-Myc, associated with angiogenesis, invasion, metastasis and proliferation and whose expression is generally increased following exposure to IR; this down-regulation was observed in the group treated with only curcumin and in the group subjected to the combined treatment. In general, since these factors are regulated by NF-kB, it can be concluded that curcumin exerts its powerful radiosensitizing effect through the down-regulation of NF-kB and its transcriptional targets, also allowing to reduce the toxic effects of inflammatory nature induced by radiation in healthy tissues.

Regarding the 51-gene signature composed by DEG common to three cell lines analyzed, the Gene Ontology analysis conducted using the DAVID tool, showed that the genes were involved in cell migration, in the calcium signaling pathways and in the activation of the chromatin modification process.

Regarding the radiotherapy context, chromatin remodelling through histones acetylation and deacetylation represents attractive target for radiosensitization as this process plays a significant role in the repair mechanisms of the double strand breaks (DSB) generated by IR.

In addition, preclinical studies have found that inhibitory molecules of HAT (histone acetyltransferase) and HDAC (histone deacetylase), such as curcumin, are able to radiosensitize neoplastic cells. In fact, it has been found that curcumin is able to significantly reduce the acetylation of H3/H4 histones and to increase the demethylation of histone H3K9 at the level of the cytokines promoter regions, thus inhibiting the expression of genes for proinflammatory chemokines^33,50,51^. Several authors have reported that curcumin limits the migration/proliferation process of neoplastic breast cancer cells by inducing crosstalk between the anchoring junctions and the Wnt pathway by means of the EPR-1 factor (Early Growth Response 1), and by inhibiting the expression of several genes involved in the epithelial-mesenchymal transition (Epithelial mesenchymal transition-EMT) (such as β-catenin, Slug etc.), a process involved in cancer progression, thus suggesting its powerful anti-metastatic function^52,53^. The antitumor effect of curcumin is also related to the increase in intracellular calcium levels (Ca2+) inducing intrinsic apoptosis, as described above.

Finally, the 263 gene-signature constituted by deregulated genes common to the two tumorigenic MCF7 and MDA-MB-231 cell lines was found to consist of DEGs implicated in the cell cycle G2/M transition, in cell migration, in angiogenesis and in calcium signaling.

Indeed, various studies have found that the curcumin anti-tumor effect is due to its ability to stop the cell cycle in G2/M phase and to trigger apoptosis of neoplastic cells by increasing the expression levels of FOX proteins, caspase 3 cleaved, Fas ligand (FasL) and the reduction of expression levels of cyclin-dependent kinases^54^.

The metabolomic analysis allowed us to highlight the effects induced on metabolism by Cur-SLN in the combined treatment on the three cell lines.

In particular, the two tumorigenic cell lines MCF7 and MDA-MB-231 showed greater similarities between them in the activated metabolic pathways, compared to the non-tumorigenic MCF10A cell line, highlighting a tumor specific metabolic response. Indeed, the hierarchical clustering reveals two defined area (the upper and the downer one) where it can be observed a tendency for which the up-regulated genes of tumorigenic cell lines are down-regulated in the MF10A, and on the contrary, the down-regulated genes of tumorigenic cell lines are up-regulated in the MCF10A (Fig. 6A). Specifically, as evidenced by the two metabolic enrichment plots, in the two tumorigenic cell lines MCF7 and MDA-MB-231, undergoing 2 Gy or 2 Gy/Cur-SNL treatments, an increase in the levels of metabolites involved in the anti-oxidant response is observed (glutathione metabolism (GSH), metabolism of arginine and proline, the pentose phosphate (PPP) pathway and the metabolism of taurine and hypotaurine), as well as of the metabolites involved in the metabolism of fatty acids and nucleotides, compared to the non-tumorigenic MCF10A cell line under the same conditions (Fig. 6B). In contrast, the same tumorigenic cell lines (MCF7 and MDA-MB-231), subjected to 2 Gy and 2 Gy/Cur-SNL treatments, showed significantly reduced levels of metabolites involved in amino acid metabolism, Krebs cycle (TCA) and glycolysis compared to the non-tumorigenic MCF10A cell line under the same treatment conditions (Fig. 6C).

Furthermore, in order the specifically highlight the radiosensitizing effect of Cur-SLN on each cell line tested, the comparison of metabolic profilings deriving from 2 Gy *vs* 2 Gy + 2.5 µM Cur-SLN has been analysed. In MCF10A cells, a group of metabolites significantly involved in amino acid metabolism were found to be significantly deregulated, showing a significant increase in glucose and citrate (Fig. 7A). In contrast, in the MDA-MB-231 cell line the significantly deregulated metabolites are involved in the metabolism of amino acids and in antioxidant processes, such as metabolism taurine and ipotaurine, fatty acids and the Krebs cycle (TCA) (Fig. 7C). Finally, consistently with the results observed in MDA-MB-231, also for MCF7 BC cells the comparison 2GY vs 2 Gy/Cur-SLN showed a significant increase in metabolites involved in the metabolism of amino acids, in antioxidant processes, in the metabolism of fatty acids and Krebs cycle (TCA), probably due to the activation of the autophagy mechanism (Fig. 7B). Then, the metabolomics analysis, in addition to the quantification of oxidative stress, has revealed that the administration of Curc-SNL on BC tumor cell lines exposed to irradiation with 2 Gy of photon beam, is able to play a protective role against oxidative stress induced by ionizing radiation and, at the same time, in the opposite direction, to induce an antitumor effect through activation of the autophagic mechanism^38^.

Based on the findings, the results obtained from this work have corroborated the literature knowledge on the wide variety of curcumin bioactivity that justify its suggested administration in combination with other strategies such as radiotherapy. As curcumin is a natural molecule with potent anti-inflammatory properties, its co-administration during the course of a fractioned protocol of irradiation with conventional radiotherapy, should enhance the cell killing effect by protecting the closed area by the onset of normal tissue complications which have inflammatory basis^55^.

Then, the formulation of a delivery system to increase the molecule stability and bio-distribution was the main aim of this study and a crucial step to move on preclinical studies and clinical trials. In conclusion, here we have shown the safety and efficacy of a curcumin delivery system (Cur-SLN) as radiosensitizer on three breast cell lines, deeply describing its molecular and metabolic effects in combined treatments.

## Conclusion

In conclusion, the data here described indicate that SLN-curcumin exerts a radiosensitizing effect, rising with its concentration increasing, particularly, Dose Modifying Factors (DMFs), calculated at the isoeffect of SF = 50%, showed that the Luminal A MCF7 resulted more sensitive to the combined treatments using increasing concentration of vehicled curcumin Cur-SLN, reaching a DMF value of 1,78 using 10 µM of Cur-SLN. Instead, the more aggressive triple negative MDA-MB-231 cells were more sensitive to free-Cur, reaching the maximum of radiosensitizer effect using 10 µM of free-Cur (DMF = 1,72). However, a clinical administration of curcumin is likely in the vehicle form, then the aggressive triple negative MDA-MB-231 BC cell line could receive a radiosensitization effect by Cur-SLN, as the DMF value is 1.38 with 10 µM of Cur-SLN. In addition, the oxidative stress quantification and the “omic” study explained the radiosensitizing function of Cur-SLN, confirming at the transcriptomic level many action mechanisms already known for curcumin, which underline anti-oxidant and anti-tumor effects, thanks to its ability to regulate various cellular processes. Moreover, also the metabolomic study highlights, in synthesis, this double action of curcumin, which on the one hand activates an anti-oxidant metabolism with protective role against IR and, on the other hand, exerts an anti-tumor role, stimulating autophagy.

Therefore, the use of curcumin loaded-SLN can be suggested in future preclinical studies and clinical trials to further test the clinical implications for the loaded curcumin concomitant use in the course of fractionated radiotherapy treatments, with the double implications of being a radiosensitizing molecule against cancer cells, with protective effects against the onset of normal tissue complications.

## Supplementary information

Supplementary info

51 gene signature.

263 gene signature

## References

[1] Baskar, R., Dai, J., Wenlong, N., Yeo, R. & Yeoh, K.W, Biological response of cancer cells to radiation treatment. *Front Mol Biosci*. 1–24 (2014). 10.3389/fmolb.2014.00024 PMC4429645 25988165

[2] Smith BD (2018). Radiation therapy for the whole breast: Executive summary of an American Society for Radiation Oncology (ASTRO) evidence-based guideline. Pract Radiat Oncol..

[3] Bartelink H (2007). Impact of a higher radiation dose on local control and survival in breast-conserving therapy of early breast cancer: 10-year results of the randomised boost versus no boost. EORTC 22881-10882 trial. J Clin Oncol..

[4] Wong P (2012). Ductal carcinoma *in situ*–the influence of the radiotherapy boost on local control. Int J Radiat Oncol Biol Phys..

[5] Meattini I (2013). Role of radiotherapy boost in women with ductal carcinoma *in situ*: a single-center experience in a series of 389 patients. Eur J Surg Oncol..

[6] Sahebkar A, Serbanc MC, Ursoniuc S, Banach M (2015). Effect of curcuminoids on oxidative stress: A systematic review and meta-analysis of randomized controlled trials. J.Funct. Foods..

[7] Aggarwal, B. B., Surh, Y. J. & Shishodia, S. The molecular targets and therapeutic uses of curcumin in health and disease. Ed. Springer. New York, ISBN-13:978-0-387-46400-8 (2007).

[8] Jagetia GC (2007). Radioprotection and radiosensitization by curcumin. Adv Exp Med Biol..

[9] Chendil D, Ranga RS, Meigooni D, Sathishkumar S, Ahmed MM (2004). Curcumin confers radiosensitizing effect in prostate cancer cell line PC-3. Oncogene..

[10] Hsu, F. T., Liu, Y. C., Liu, T. T. & Hwang, J. J. Curcumin Sensitizes Hepatocellular Carcinoma Cells to Radiation via Suppression of Radiation-Induced NF-κB Activity. *Biomed Res Int*. 363671 (2015). 10.1155/2015/363671 PMC4619792 26539482

[11] Shishodia S, Amin HM, Lai R, Aggarwal BB (2005). Curcumin (diferuloylmethane) inhibits constitutive NF-kappaB activation, induces G1/S arrest, suppresses proliferation, and induces apoptosis in mantle cell lymphoma. Biochem Pharmacol..

[12] Qiao Q, Jiang Y, Li G (2013). Inhibition of the PI3K/AKT-NF-κB pathway with curcumin enhanced radiation-induced apoptosis in human Burkitt’s lymphoma. J Pharmacol Sci..

[13] Li M, Zhang Z, Hill DL, Wang H, Zhang R (2007). Curcumin, a dietary component, has anticancer, chemosensitization, and radiosensitization effects by down-regulating the MDM2 oncogene through the PI3K/mTOR/ETS2 pathway. Cancer Res..

[14] Ibrahim A (2011). Curcumin induces apoptosis in a murine mammary gland adenocarcinoma cell line through the mitochondrial pathway. Eur J Pharmacol..

[15] Khan N, Afaq F, Mukhtar H (2007). Apoptosis by dietary factors: the suicide solution for delaying cancer growth. Carcinogenesis..

[16] Okunieff P (2006). Curcumin protects against radiation-induced acute and chronic cutaneous toxicity in mice and decreases mRNA expression of inflammatory and fibrogenic cytokines. Int J Radiat Oncol Biol Phys..

[17] Bravatà V, Cammarata FP, Forte GI, Minafra L (2013). “Omics” of HER2-positive breast cancer. OMICS..

[18] Amore E (2017). Mucoadhesive solid lipid microparticles for controlled release of a corticosteroid in the chronic obstructive pulmonary disease treatment. Nanomedicine (London)..

[19] Bravatà V (2015). Gilardi, M.C., High-dose Ionizing Radiation Regulates Gene Expression Changes in the MCF7 Breast Cancer Cell Line. Anticancer Res..

[20] Minafra L (2015). Gene Expression Profiling of MCF10A Breast Epithelial Cells Exposed to IOERT. Anticancer Res..

[21] Militello C (2017). Area-based cell colony surviving fraction evaluation: A novel fully automatic approach using general-purpose acquisition hardware. Comput Biol Med..

[22] Medhora M (2012). Dose-modifying factor for captopril for mitigation of radiation injury to normal lung. J Radiat Res..

[23] International Atomic Energy Agency: Adsorbed Dose Determination in External Beam Radiotherapy. An International Code of Practice for Dosimetry Based on Standards of Absorbed Dose to Water. *Technical Reports Series* No. 398, Vienna, (2000).

[24] Barrett T (2013). NCBI GEO: archive for functional genomics data sets–update. Nucleic Acids Res..

[25] Gaglio D (2011). Oncogenic K-Ras decouples glucose and glutamine metabolism to support cancer cell growth. Mol Syst Biol..

[26] Bondì ML (2017). Biocompatible Lipid Nanoparticles as Carriers To Improve Curcumin Efficacy in Ovarian Cancer Treatment. J Agric Food Chem..

[27] Hagl S (2015). Curcumin micelles improve mitochondrial function in neuronal PC12 cells and brains of NMRI mice - Impact on bioavailability. Neurochem Int..

[28] Wang JQ (2018). Novel curcumin analogue hybrids: Synthesis and anticancer activity. Eur J Med Chem..

[29] Wang S (2018). Synthesis of water-soluble curcumin derivatives and their inhibition on lysozyme amyloid fibrillation. Spectrochim Acta A Mol Biomol Spectrosc..

[30] Wang Z (2018). Curcumin restrains hepatic glucose production by blocking cAMP/PKA signaling and reducing acetyl CoA accumulation in high-fat diet (HFD)-fed mice. Mol Cell Endocrinol..

[31] Wilken R, Veena MS, Wang MB, Srivatsan ES (2011). Curcumin: A review of anti-cancer properties and therapeutic activity in head and neck squamous cell carcinoma. Mol Cancer..

[32] Gan L, Li C, Wang J, Guo X (2016). Curcumin modulates the effect of histone modification on the expression of chemokines by type II alveolar epithelial cells in a rat COPD model. Int J Chron Obstruct Pulmon Dis..

[33] Hu J (2017). Curcumin modulates covalent histone modification and TIMP1 gene activation to protect against vascular injury in a hypertension rat model. Exp Ther Med..

[34] Collins HM (2013). Differential effects of garcinol and curcumin on histone and p53 modifications in tumour cells. BMC Cancer..

[35] Wang J, Wang C, Bu G (2018). Curcumin inhibits the growth of liver cancer stem cells through the phosphatidylinositol 3-kinase/protein kinase B/mammalian target of rapamycin signaling pathway. Exp Ther Med..

[36] Kato M (2017). Curcumin improves glucose tolerance via stimulation of glucagon-like peptide-1 secretion. Mol Nutr Food Res..

[37] GUO SHOUYU, LONG MINGZHI, LI XIUZHEN, ZHU SHUSHU, ZHANG MIN, YANG ZHIJIAN (2016). Curcumin activates autophagy and attenuates oxidative damage in EA.hy926 cells via the Akt/mTOR pathway. Molecular Medicine Reports.

[38] Kim HS, Luo L, Pflugfelder SC, Li DQ (2005). Doxycycline inhibits TGF-beta1-induced MMP-9 via Smad and MAPK pathways in human corneal epithelial cells. Invest Ophthalmol Vis Sci..

[39] Park S, Cho DH, Andera L, Suh N, Kim I (2013). Curcumin enhances TRAIL-induced apoptosis of breast cancer cells by regulating apoptosis-related proteins. Mol Cell Biochem..

[40] Saini S (2011). Curcumin modulates microRNA-203-mediated regulation of the Src-Akt axis in bladder cancer. Cancer Prev Res (Phila)..

[41] Jia T (2014). The differential susceptibilities of MCF-7 and MDA-MB-231 cells to the cytotoxic effects of curcumin are associated with the PI3K/Akt-SKP2-Cip/Kips pathway. Cancer Cell Int..

[42] Cheng C (2016). Curcumin induces G2/M arrest and triggers apoptosis via FoxO1 signaling in U87 human glioma cells. Mol Med Rep..

[43] Jain A (2015). Curcumin inhibits PhIP induced cytotoxicity in breast epithelial cells through multiple molecular targets. Cancer Lett..

[44] Han J (2012). Curcumin induces autophagy to protect vascular endothelial cell survival from oxidative stress damage. Autophagy..

[45] Xiang L (2011). Tetrahydrocurcumin extends life span and inhibits the oxidative stress response by regulating the FOXO forkhead transcription factor. Aging (Albany NY)..

[46] Lin A (2014). FoxO transcription factors promote AKT Ser473 phosphorylation and renal tumor growth in response to pharmacological inhibition of the PI3K-AKT pathway. Cancer Res..

[47] Firat E, Niedermann G (2016). FoxO proteins or loss of functional p53 maintain stemness of glioblastoma stem cells and survival after ionizing radiation plus PI3K/mTOR inhibition. Oncotarget..

[48] Kunnumakkara AB, Anand P, Aggarwal BB (2008). Curcumin inhibits proliferation, invasion, angiogenesis and metastasis of different cancers through interaction with multiple cell signaling proteins. Cancer Lett..

[49] Li X (2015). Proteomic analyses reveal distinct chromatin-associated and soluble transcription factor complexes. Mol Syst Biol..

[50] Oike T (2012). Garcinol, a histone acetyltransferase inhibitor, radiosensitizes cancer cells by inhibiting non-homologous end joining. Int J Radiat Oncol Biol Phys..

[51] Chen QY (2015). Curcumin inhibits proliferation-migration of NSCLC by steering crosstalk between a Wnt signaling pathway and an adherens junction via EGR-1. Mol Biosyst..

[52] Zhang Z (2016). Curcumin inhibits tumor epithelial-mesenchymal transition by downregulating the Wnt signaling pathway and upregulating NKD2 expression in colon cancer cells. Oncol Rep..

[53] Imran M (2018). Cucurmin, anticancer, & antitumor perspectives: A comprehensive review. Crit Rev Food Sci Nutr..

[54] Forte GI (2017). Radiogenomics: the utility in patient selection. Transl Cancer Res..

[55] West, C. M. & Barnett, G. C. Genetics and genomics of radiotherapy toxicity: towards prediction. *Genome Medicine.* **3**, 52 (2011). 10.1186/gm268 PMC3238178 21861849

