# Supplementary material for: Radiosensitizing effect of curcumin-loaded lipid nanoparticles in breast cancer cells
Source: Sci Rep. 2019 Jul 31;9:11134. doi: 10.1038/s41598-019-47553-2 (PMC6668411; doi:10.1038/s41598-019-47553-2)
Supplement: Supplementary file 1 — Supplementary info [file 41598_2019_47553_MOESM1_ESM.pdf]

Radiosensitizing effect of curcumin-loaded lipid nanoparticles in breast cancer cells

Luigi Minafra[1*], Nunziatina Porcino[1*], Valentina Bravatà[1§], Daniela Gaglio[1,2], Marcella Bonanomi[2], Erika Amore[3], Francesco Paolo Cammarata[1], Giorgio Russo[1], Carmelo Militello[1], Gaetano Savoca[1], Margherita Baglio[1], Boris Abbate[4], Giuseppina Iacoviello[4], Giovanna Evangelista[5], Maria Carla Gilardi[1,6], Maria Luisa Bondì[3], Giusi Irma Forte[1]

[1]Istituto di Bioimmagini e Fisiologia Molecolare-Consiglio Nazionale delle Ricerche (IBFM-CNR), Cefalù (PA), Italy

[2]SYSBIO Centre of Systems Biology, University of Milano-Bicocca, Milano, Italy.

[3]Istituto per lo Studio dei Materiali Nanostrutturati-Consiglio Nazionale delle Ricerche (ISMN-CNR), Palermo, Italy

[4]Medical Physics Department, ARNAS-Civico Hospital, Palermo, Italy.

[5]Radiation Oncology, ARNAS-Civico Hospital, Palermo, Italy.

[6]Department of Medicine and Surgery, University of Milano-Bicocca, Monza, Italy

*These authors contributed equally to this work

[§]Corresponding author: PhD Valentina Bravatà: valentina.bravata@ibfm.cnr.it

email addresses:

Luigi Minafra: luigi.minafra@ibfm.cnr.it
Nunziatina Porcino: nunziatina.porcino@ibfm.cnr.it
Daniela Gaglio: daniela.gaglio@ibfm.cnr.it
Marcella Bonanomi: marcella.bonanomi@unimib.it
Erika Amore: erika.amore@pa.ismn.cnr.it
Francesco Paolo Cammarata: francesco.cammarata@ibfm.cnr.it
Giorgio Russo: giorgio.russo@ibfm.cnr.it
Carmelo Militello: carmelo.militello@ibfm.cnr.it
Gaetano Savoca: savoca.gaetano@gmail.com
Margherita Baglio: margo4@hotmail.it
Boris Abbate: borisfederico.abbate@arnascivico.it
Giuseppina Iacoviello: giuseppina.iacoviello@arnascivico.it
Giovanna Evangelista: giovanna.evangelista@arnascivico.it
Maria Carla Gilardi: mariacarla.gilardi@ibfm.cnr.it
Maria Luisa Bondì: ricercatore.bondi@gmail.com
Giusi Irma Forte: giusi.forte@ibfm.cnr.it

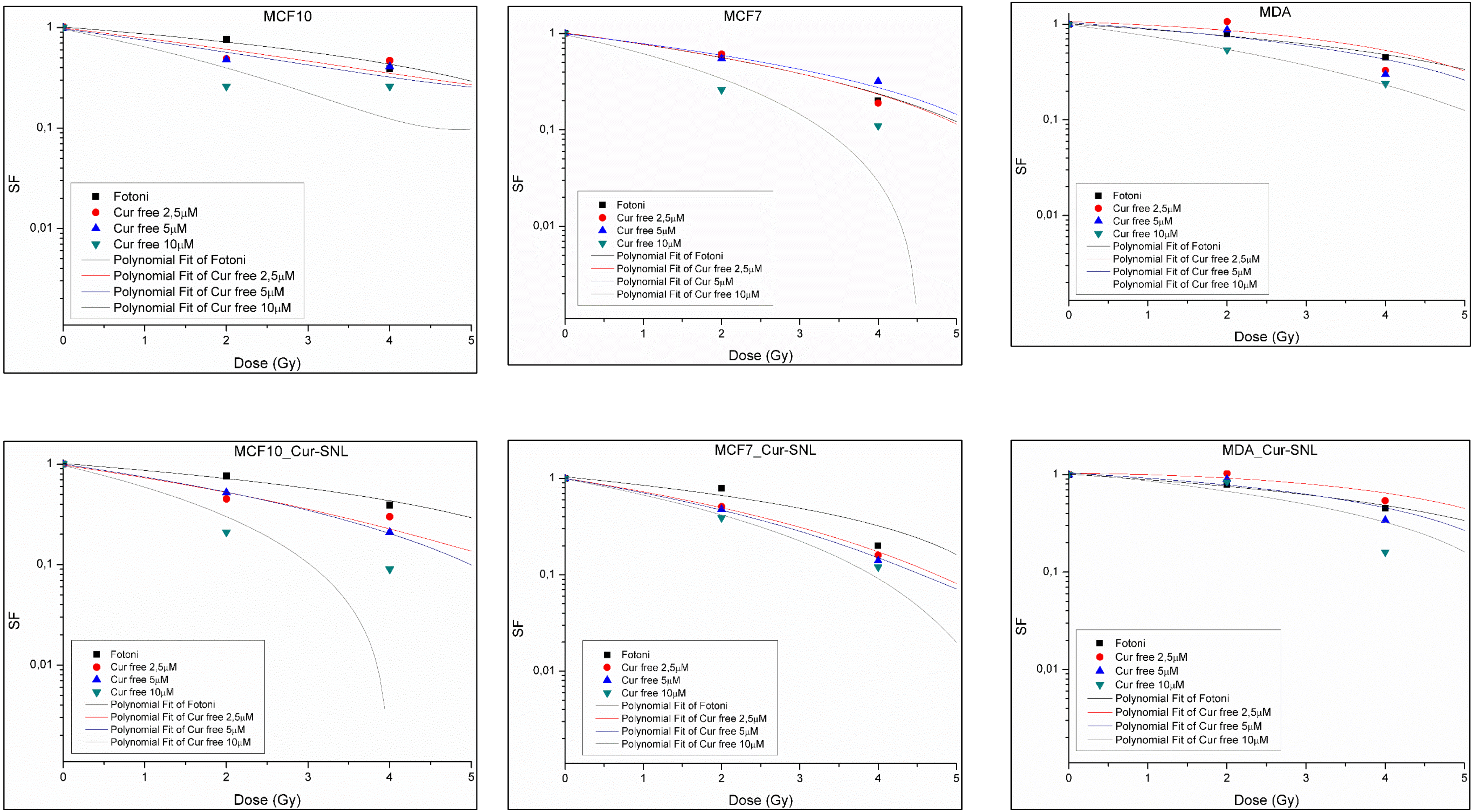
MCF10
SF
1
0,1
0,01
Dose (Gy)
0
1
2
3
4
5
Fotoni
Cur free 2,5μM
Cur free 5μM
Cur free 10μM
Polynomial Fit of Fotoni
Polynomial Fit of Cur free 2,5μM
Polynomial Fit of Cur free 5μM
Polynomial Fit of Cur free 10μM
MCF7
SF
Dose (Gy)
Fotoni
Cur free 2,5μM
Cur free 5μM
Cur free 10μM
Polynomial Fit of Fotoni
Polynomial Fit of Cur free 2,5μM
Polynomial Fit of Cur 5μM
Polynomial Fit of Cur free 10μM
MDA
SF
Dose (Gy)
Fotoni
Cur free 2,5μM
Cur free 5μM
Cur free 10μM
Polynomial Fit of Fotoni
Polynomial Fit of Cur free 2,5μM
Polynomial Fit of Cur free 5μM
Polynomial Fit of Cur free 10μM
MCF10_Cur-SNL
SF
Dose (Gy)
Fotoni
Cur free 2,5μM
Cur free 5μM
Cur free 10μM
Polynomial Fit of Fotoni
Polynomial Fit of Cur free 2,5μM
Polynomial Fit of Cur free 5μM
Polynomial Fit of Cur free 10μM
MCF7_Cur-SNL
SF
Dose (Gy)
Fotoni
Cur free 2,5μM
Cur free 5μM
Cur free 10μM
Polynomial Fit of Fotoni
Polynomial Fit of Cur free 2,5μM
Polynomial Fit of Cur free 5μM
Polynomial Fit of Cur free 10μM
MDA_Cur-SNL
SF
Dose (Gy)
Fotoni
Cur free 2,5μM
Cur free 5μM
Cur free 10μM
Polynomial Fit of Fotoni
Polynomial Fit of Cur free 2,5μM
Polynomial Fit of Cur free 5μM
Polynomial Fit of Cur free 10μM
